# Additive Manufacturing for Neurosurgery: Digital Light Processing of Individualized Patient-Specific Cerebral Aneurysms

**DOI:** 10.3390/ma14206057

**Published:** 2021-10-14

**Authors:** Stefano Guarino, Enrico Marchese, Gennaro Salvatore Ponticelli, Alba Scerrati, Vincenzo Tagliaferri, Federica Trovalusci

**Affiliations:** 1 Department of Engineering, University of Rome “Niccolò Cusano”, Via Don Carlo Gnocchi 3, 00166 Rome, Italy; stefano.guarino@unicusano.it (S.G.); gennaro.ponticelli@unicusano.it (G.S.P.); 2 Department of Neurosurgery, Catholic University of Rome, L.go A. Gemelli 8, 00100 Rome, Italy; enrico.marchese@policlinicogemelli.it; 3 Department of Transalational Medicine, University of Ferrara, Via Aldo Moro 8, 44124 Ferrara, Italy; 4 Department of Enterprise Engineering, University of Rome Tor Vergata, Via del Politecnico 1, 00133 Rome, Italy; tagliafe@uniroma2.it (V.T.); federica.trovalusci@uniroma2.it (F.T.)

**Keywords:** 3D printing, additive manufacturing, digital light processing, neurosurgery, cerebral aneurysms

## Abstract

This study aims at demonstrating the feasibility of reproducing individualized patient-specific three-dimensional models of cerebral aneurysms by using the direct light processing (DLP) 3D printing technique in a low-time and inexpensive way. Such models were used to help neurosurgeons understand the anatomy of the aneurysms together with the surrounding vessels and their relationships, providing, therefore, a tangible supporting tool with which to train and plan surgical operations. The starting 3D models were obtained by processing the computed tomography angiographies and the digital subtraction angiographies of three patients. Then, a 3D DLP printer was used to print the models, and, if acceptable, on the basis of the neurosurgeon’s opinion, they were used for the planning of the neurosurgery operation and patient information. All the models were printed within three hours, providing a comprehensive representation of the cerebral aneurysms and the surrounding structures and improving the understanding of their anatomy and simplifying the planning of the surgical operation.

## 1. Introduction

During the last few years, 3D printing technology has experienced a significant breakthrough, allowing the fabrication of very complex structures with different materials, from polymers [1] to metals [2,3] to ceramics [4,5] and composites [6,7], and for a wide range of applications, from personalized consumer products [8,9,10,11] to the manufacturing industry [12,13,14,15] and, more recently, for science [16,17,18,19], education [20,21], and clinical practice [22,23,24].

3D printing is playing a central role in transforming healthcare and clinical practice because it is allowing a remarkable enhancement in terms of patient-care thanks to the ability to realize individualized models, implants, and tools [22]. In fact, with this technology, any kind of structure can be reconstructed from 3D images and subsequently fabricated as 3D physical models.

In this context, clinical medicine, and in particular neurosurgery [25,26,27], is undergoing an unprecedented improvement, making surgeons able to intervene in individualized, patient-based, and, if necessary, timely ways [28]. Moreover, a 3D model can be used for surgical planning and education for both patients and trainees, as well as enabling surgical devices to be designed and fabricated for personalized treatments [29].

The field of neurosurgery has experienced substantial progress as a result of the usage of 3D printing. Studies involving the incorporation of 3D printing in neurosurgery have focused upon three main areas: (i) the creation of patient-specific anatomical models for surgical planning, training, and education; (ii) the design of neurosurgical devices for the assessment and treatment of neurosurgical diseases; and (iii) the development of biological tissue-engineered implants. In neuro-oncology, 3D printing technology has enabled MRI data to be translated into patient-specific models depicting the associations between a tumour, the skull, vasculature, and surrounding non-pathologic brain tissue [30,31], or for the development of simulators created from a multitude of materials with varying consistencies and densities [32]. In functional neurosurgery, printed moulds from personalized silicone sheets with embedded electrodes can be produced with 3D printing [33]. For spinal surgery, a patient-specific screw guide that locks onto the lamina to prevent erroneous movement has been proposed [34]. Moreover, ventriculostomy has been widely studied as a procedure suitable for 3D printing applications [35].

The main impact of 3D printing in neurosurgery concerns vascular neurosurgery, mainly for preoperative planning and surgery simulations, because neurosurgeons often come across surgical procedures that involve very intricate anatomical structures [28].

Before the advent of 3D printing, neuroimaging has enabled the visualization of complex anatomical structures, especially through computed tomography, magnetic resonance imaging, and X-ray [36], but with the limitation of acquiring 2D images [37], therefore preventing a full comprehension of the relationships between the structures involved because neurosurgeons have to mentally reconstruct them in a 3D view [38]. More recently, the development of computed tomography angiography (CTA) and digital subtraction angiography (DSA) have allowed the reconstruction of the anatomical structure of interest, e.g., cerebral aneurysms, in a 3D computational image. However, even if neurosurgeons can better understand the anatomical details of the aneurysms, these are viewed on a 2D flat computer screen, thus making interpretations related to depth quite difficult [28]. In this light, 3D printing has appeared as an innovative potential tool with the ability to replicate even highly intricate anatomical structures [39].

The first work aimed at reproducing intracranial aneurysms dates back to 1999, when D’Urso et al. [40] reported on the use of a stereolithography apparatus to print the cerebrovascular structures of 19 patients. The surgeons involved in the study declared that the 3D printed models reproduced the intraoperative findings faithfully. However, perforating vessels with a thickness smaller than 1 mm were not replicated. Moreover, the models were printed with an average time of 3 days and a cost of around €250. Then, in 2004, Wurm et al. [41] used a stereolithography apparatus to reproduce the structures of cerebral aneurysms with the parent and surrounding vessels of 13 patients based on CTAs and DSAs. The surgeons involved reported that the 3D printed models accurately replicated the intraoperative findings. The main drawbacks highlighted in that study were the time needed to fabricate and deliver the models, and that the rigidity of the material did not allow dissecting exercises to be performed. In 2009, Kimura et al. [42] reproduced the first hollow aneurysms models with a rubber-like polymer through stereolithography. The manufacturing process took 3 to 7 days to complete, and the cost was between €250 and €340 per patient according to the size and complexity of the aneurysm. Moreover, the thickness and consistency of the vessel walls were not precisely replicated and negatively affected accuracies in clipping. Afterwards, Wurm et al. [43], in 2011, improved their technique with the use of a multimaterial stereolithography 3D printer. In particular, the aneurysm and its parent vessels were produced from a rubber-like flexible material in order to have a more realistic model. However, the material was still too rigid and brittle to simulate human vessel consistency. Some years later, in 2015, Mashiko et al. [38] reproduced hollow silicone models of 20 patients using 3D printed aneurysms as base models in only several hours. However, inaccuracies, such as discrepancies in vessel wall thickness, elasticity, and adhesion, were the most significant limitations. In the same year, Anderson et al. [44] replicated the cerebral aneurysms of 10 patients using a fused deposition modelling-based printer with polylactic acid, showing that even low-cost desktop 3D printers can fabricate accurate models of intricate anatomical structures, such as cerebral aneurysms, in a cost-effective and timely manner.

During the last few years, many technological improvements have been made for both 3D printers and materials [45,46], allowing a significant reduction of the related costs [17] and enhancing the final quality of the 3D printed models [47]. However, 3D printing technology still presents some limitations if compared to the most advanced neuroimaging methods, i.e., CTA and DSA, since these are commonly available in most hospitals and image processing takes a few minutes. On the other hand, high-accuracy 3D printers are still quite expensive and are usually not supplied in hospitals [48]. Moreover, the mean time to obtain a 3D printed model is definitely longer than the conventional imaging [25,38,49,50], and vessels with a diameter smaller than 1 mm tend to be underestimated and cannot be correctly reproduced [40].

In this context, the present study aimed at proposing the direct light processing 3D printing technique for the reproduction of cerebral aneurysms. The optimization of the process focused on the reduction of the time to obtain the final model, starting from the elaboration of CTAs or DSAs, and of the relative production costs, ensuring at the same time high quality and accuracy by printing vessels with a diameter smaller than 1 mm. Moreover, this work aimed to highlight the ability of the 3D printed aneurysm models to support neurosurgeons regarding surgical planning and patient information, improving the anatomy knowledge on an individualized, patient-specific basis.

## 2. Materials and Methods

**Figure 1:**
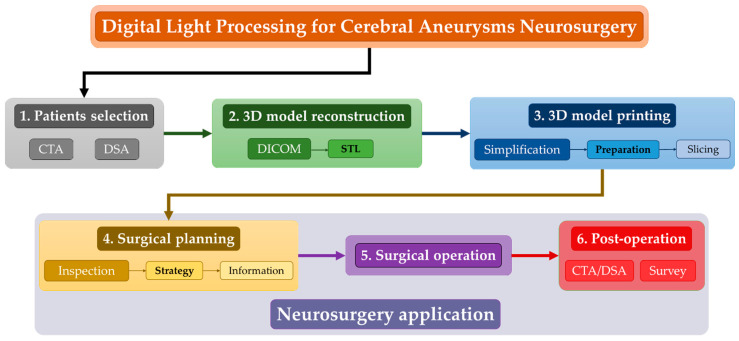
Flowchart of the activities carried out for the reproduction of the cerebral aneurysms through the digital light processing 3D printing technique and their subsequent use for the surgical planning and operation. CTA: computer tomography angiography; DSA: digital subtraction angiography; DICOM: Digital Imaging and Communication in Medicine; STL: Standard Triangulation Language.

The aim of the study is to assess the feasibility of the direct light processing 3D printing technique to reproduce, in a low-time, inexpensive, and accurate way, the anatomical structure of cerebral aneurysms and the surrounding vessels for neurosurgeon training and surgical operation planning. To this end, the activity concerned six main steps, as shown in Figure 1 and detailed in the next sections. 

The following is a short summary:

(1) Firstly, among the patients hospitalized at the Department of Neurosurgery of Policlinico A. Gemelli of Rome, three of them with a cerebral aneurysm agreed to participate in the investigation; for them, a computer tomography angiography (CTA) and a digital subtraction angiography (DSA) were performed;

(2) During the second step, the 3D models of the cerebral aneurysms and the surrounding vessels were reconstructed by using the commercial software 3DSlicer^®^. This step started from the data output of the CTAs and DSAs as Digital Imaging and Communication in Medicine (DICOM) and exporting the reconstructed 3D models as Standard Triangular Language (STL) file;

(3) The third step concerned the 3D DLP printing process by using the SHAREBOT Voyager 2 printer; this step involved the neurosurgeons for the simplification of the model in order to speed up the printing process for which it was properly prepared by choosing the appropriate support structure and finally sliced;

(4) After printing, the 3D model of each patient was inspected in order to define the best strategy for the surgical operation and further used to inform the patient about the chosen plan;

(5) Once the patient’s approval to perform the operation was obtained, the neurosurgeons carried out the needed treatment by adopting the planned strategy;

(6) Finally, after the operations, CTAs or DSAs were collected in order to verify the success of the treatment. Moreover, the neurosurgeons who planned and performed the surgery were involved in a survey to evaluate the utility of the 3D printed models.

### 2.1. Patients Selection

The patients who agreed to participate in the investigation are three women whose main information and aneurysms characteristics are summarized in Table 1.

For patient #1, only a CTA was performed, while, for the other two patients, both the CTA and DSA. The angiographic files were acquired using the monoplane angiography equipment Neurostar TOP by Siemens with a rotational angiography greater than 200° every 4° and at a rate of 10 frames per second.

### 2.2. 3D Model Reconstruction

**Figure 2:**
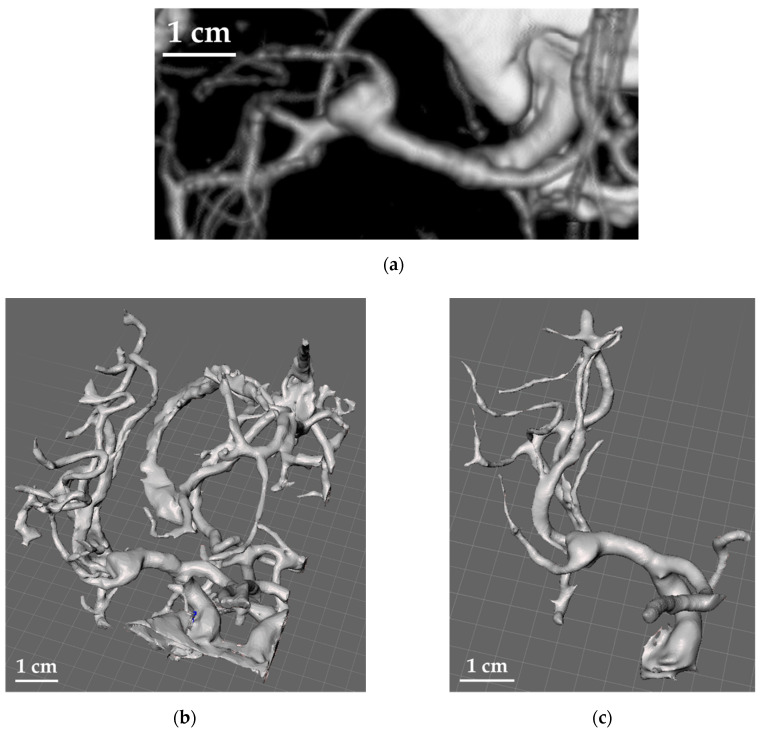

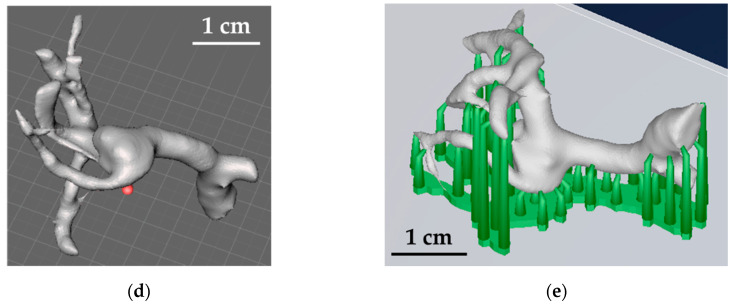
Elaboration of the STL file starting from the DICOM image of a cerebral aneurysm structure: (**a**) CTA image; (**b**) conversion to the first STL file; (**c**) first simplification of the model; (**d**) second simplification of the model in order to be limited to the surrounding of the aneurysm to speed up the printing process; (**e**) creation of the supports (green structures) and slicing.

For the reconstruction of the 3D models of the aneurysms and the surrounding vessels structures, the software 3DSlicer^®^ was used to extract the data of the CTAs and DSAs as DICOM images (Figure 2a) and convert them into the final STL files to be used for printing (Figure 2b).

Each image was segmented to the particular threshold of the tissue to be modelled. In particular, the principle on which this technique is based is the definition of a reference threshold and the assignation of a “label” to each pixel of the grayscale image. Such labels will declare whether the relative pixel belongs to the region of interest or not. The decision criterion is, therefore, associated with the crossing of the defined intensity threshold, starting from the strong assumption that the searched structure has a generally different intensity value compared to the background pixels. Despite their simplicity, these methods allow good results to be obtained by the extraction of the tissues of interest from the images obtained by diagnostic methods, such as CTA and DSA, because they exploit the absolute nature of the intensity values recorded, which represent the values of the radiation permeability of tissues, measured in ‘Hounsfield Units’ [51,52]. Specifically, only the pixels characterized by a grey value included within the selected threshold range were considered. Moreover, the trilinear grey value interpolation was used to generate the contours from each image and the contours “in between” to match the resolution of the Z-axis of the 3D printer adopted set at 10 μm. This process was carried out both manually and automatically in order to compare the time and resolution that can be achieved for the two different approaches.

The results were converted to an STL file, and the obtained models were further edited to remove non-essential structures with the help of the neurosurgeons by using the software Meshmixer^®^ (by Autodesk, San Rafael, CA, USA), Figure 2c,d. In fact, since the manufacturing time is governed by the number of layers to be printed, it was necessary to minimize the size of the aneurysm along the Z-axis (i.e., the printing direction). After the appropriate simplifications, the support structures necessary for the printing process were properly added, and, finally, the models were sliced in the optimal build orientation with the software Pyramis^®^ (by CIMsystem, Milan, Italy), Figure 2e. In this case, for the support structure, pillars with circular sections were used with connection pins reduced to a minimum diameter of about 0.3 mm, able to guarantee a good connection with the model and, at the same time, easy removal. These were added manually to avoid the piercing of the pillars through the branches of the aneurysm and to ensure that they were enough to efficiently support the complex structure of the model.

### 2.3. 3D Model Printing

The third activity (see Figure 1) concerned the fabrication of the 3D models by using the direct light processing 3D printer Voyager 2 by SHAREBOT, whose main characteristics are reported in Table 2. The material used is the ABS-like photoactive resin Share-HT by SHAREBOT, made of 70–80% of aliphatic acrylates and 20–30% of urethane acrylate with a resulting density of around 1 g/cm^3^. Table 3 reports the main mechanical properties of the material as declared by the producer.

It is worth highlighting that a preliminary investigation was carried out in order to find the optimal process parameters in order to guarantee both quality and rapidity to reproduce the final model. In particular, among the operational parameters, most of which are imposed by the 3D printer producer, the layer height (i.e., Z-resolution) and curing time were optimized.

After printing, in order to evaluate the accuracy of reproducing the starting aneurysm model, a coordinate measurement machine by Hexagon was adopted to measure the main dimensions of the thicker parts of the models and to calculate the actual shrinkage. It is worth noting that it was not possible to measure the smallest branches due to the size of the contact probe and the difficulty of reaching specific positions due to the very complex geometry of the aneurysms.

### 2.4. Neurosurgery Application

After printing, the 3D models were given to the neurosurgeons to let them inspect the anatomical structure of the cerebral aneurysms and plan the surgical strategy in terms of type of clipping, whose goal is to isolate the aneurysm from the normal circulation without blocking off any small perforating arteries nearby, and to inform the patient on the chosen treatment.

In order to verify the improved anatomical knowledge of the aneurysm and the subsequent established strategy by using the 3D printed model, the planning of the surgical clipping was previously defined based only on the standard imaging techniques.

The model was then sterilized in order to be used within the surgery room during the operation.

In order to verify the success of the treatment and evaluate the presence of any residual neck of the aneurysms, post-operative CTAs or DSAs were collected.

Finally, after the surgery, in order to qualitatively investigate the usefulness of the 3D printed models, surgeons were asked to rate the models for reliability on a scale from 0 to 5.

## 3. Results and Discussion

Anatomy knowledge of the aneurysm, of the surrounding vascular structures and how they are connected to each other, is of crucial importance in neurosurgery. In fact, neurosurgeons need a complete and the most confident comprehension of the whole structure and interconnections to define the best strategy for the operation.

In this context, the 3D printing technology can be considered a valid support for the improvement of the anatomy knowledge, as well as for the training and the surgical planning thanks to the ability to very accurately reproduce even the complex structure that typically characterizes a cerebral aneurysm and its surroundings. This makes the 3D printed model replica of the patient’s aneurysm a new tool that can be used within the surgery room to help the neurosurgeons choose the best strategy and to modify it if necessary, in this way avoiding potential damage to the surrounding vascular structure.

### 3.1. 3D Printing

One of the main goals of the study was to reproduce the 3D models of the cerebral aneurysms in a suitable time in order to guarantee the availability of the final model in the case of an emergency. In fact, the mean time between the CTA or DSA acquisition and the beginning of the surgery, as declared by the Department of Neurosurgery of the Policlinico A. Gemelli, is about 150 min. For this reason, a preliminary activity concerned the optimization of the DLP process by finding the best combination of the Z-resolution (i.e., layer height) and curing time (i.e., exposing time of the resin to the UV light) able to guarantee quality and rapidity. In this case, a layer height of 10 μm and a curing time of 1.5 s for each layer were chosen. This combination allowed printing a minimum vessel diameter of 0.5 mm. However, no post-curing process was performed in order to make the 3D models available in a shorter time. Further, after printing, a manual removal of the supports was necessary.

Table 4 reports the times needed to perform all the steps involved in the reproduction and printing of the 3D models of the cerebral aneurysms: the time for the elaboration of the STL file by manually defining the images’ contours from the DICOM files ($t_{man}^{STL}$); the time for the elaboration of the STL file by automatically defining the images’ contours from the DICOM files ($t_{aut}^{STL}$); the time for the printing process ($t_{P}$); and the time needed for the preparation and slicing of the 3D models within the slicing software and for the manual removal of the support structures ($t_{S}$). The sum of these contributions leads, respectively, to the manual and automatic elaboration of the DICOM files to the total times for the completion of the entire process $t_{man}^{Tot}$ and $t_{aut}^{Tot}$.

**Figure 3:**
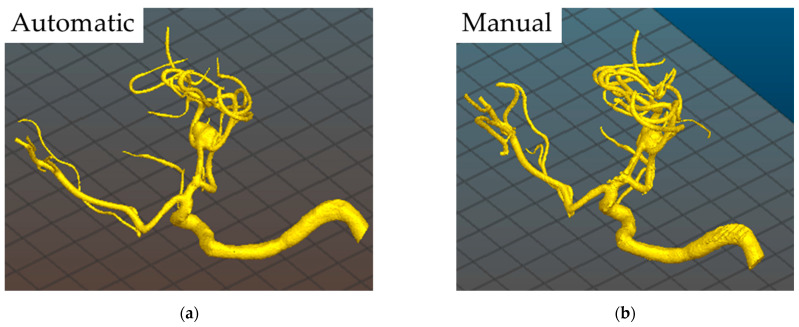
Comparison of reconstruction methods for the reproduction of the images’ contours for the elaboration of the STL file from the DICOM data: (**a**) automatic contour; (**b**) manual contour.

In general, the elaboration of the CTAs or DSAs through the automatic process is quicker, but it sometimes results in a lower definition, especially for CTAs, requiring the performance of a manual evaluation of the images’ contours (see Figure 3). The suggestion is, therefore, to perform a manual elaboration for the CTAs and an automatic elaboration for the DSAs. Moreover, it is worth noting that both the time needed for the elaboration of the STL file (i.e., $t_{man}^{STL}$) and for the preparation of the 3D model for printing and post-processing operations (i.e., $t_{S}$) strongly depends on the operator’s experience.

The mean total time was about 183 min and 170 min considering the manual or automatic processing of the STL files, respectively. These values can be considered very interesting and also absolutely suitable for emergency use in the case of ruptured aneurysms, which need around 150 min to be ready for surgery. In this way, the 3D printed model could be available for the neurosurgeons just before they reach the brain and the aneurysm site, thus allowing better planning of the clipping.

Regarding the production costs (see Table 5), the analysis revealed an average cost per model of about €1.19 without considering the labour cost. The result is comparable to the studies in the literature [25,38,48,49,50], as shown in Table 6 where a production cost of lower than 23 €/model is reported depending on the material and the printer adopted and the anatomic structure reproduced. In fact, in this study, only the aneurysms with a small part of the vascular tree were reproduced, while, in the cited works, skulls and brains were also printed, and a silicon-based hollow reproduction was made, thus explaining both the longer times, more than 4 h at least, and higher costs. Despite this, the approach proposed here can be considered suitable for printing 3D models of cerebral aneurysms in a timely and inexpensive manner, making the models available also for emergency cases of ruptured aneurysms.

Finally, the DLP machine adopted here allowed printing the 3D models of the cerebral aneurysms with an average shrinkage of around 0.552%, which was evaluated on the thicker parts of the models. This result is in accordance with the typical values found in the literature [48] and highlights the ability of this technique to reproduce, in a highly accurate way, even very intricate structures as cerebral aneurysms.

### 3.2. Neurosurgery Application

The strategy to be adopted generally consists of the definition of the types and number of clips as well as the way they should be applied. Table 7 reports the comparison between the adopted clips and the planned ones with the use of the 3D printed models as supports. As a result, there was a very high correspondence between the choice made during the preoperative planning with the 3D models and the real clipping used during the surgery. It is worth highlighting that only using the imaging techniques was not enough to discriminate between single or multiple clipping due to the difficulty to understand if the surrounding vessels were detachable or tightly adhered, especially for patients #1 and #2. The 3D printed models helped to better understand such a relationship and propose a more specific approach, as detailed in Table 7. No residual neck was observed after the surgery for all the patients, thus suggesting a complete success of the planned treatment.

**Figure 4:**
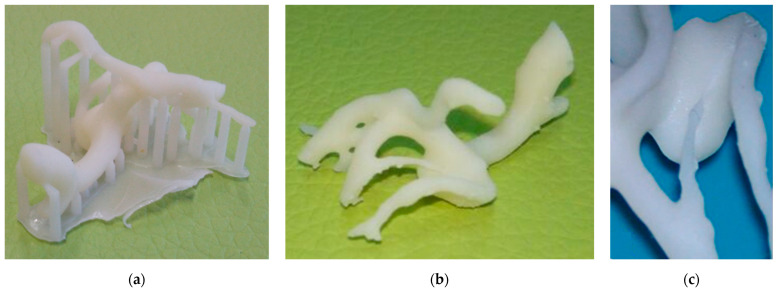
Patient #1 aneurysm reproduction: (**a**) 3D printed model showing the support structures; (**b**) 3D printed model after the removal of the support structures; (**c**) a particular of the back side of the aneurysm showing a possible adherence between the dome of the aneurysm and the frontal branch.

The 3D model reconstruction of the aneurysm of patient #1 (i.e., unruptured 5 mm L-MCA aneurysm) showed a wide neck and a possible adherence between the dome of the aneurysm and the frontal branch (see Figure 4). A multiple clipping was suggested. The patient underwent surgery with a multiple clipping as planned.

**Figure 5:**
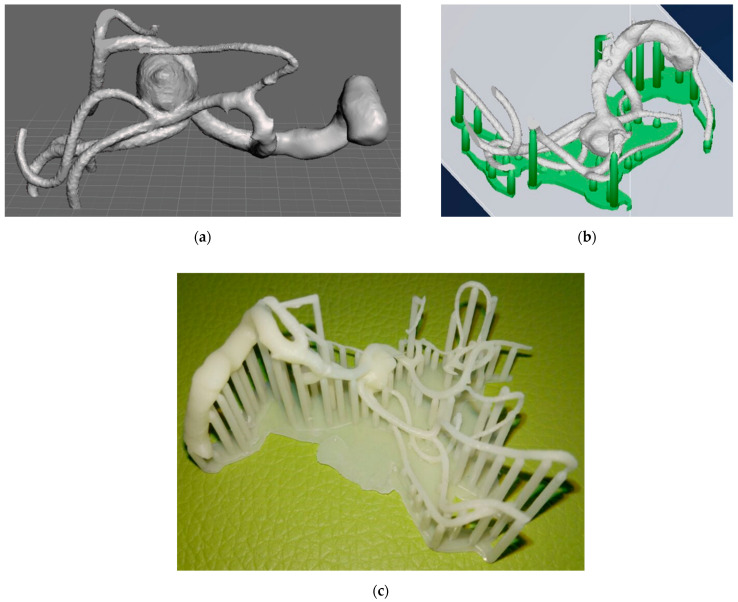
Patient #2 aneurysm reproduction and printing: (**a**) 3D model reconstruction of the aneurysm after simplification suggested by the neurosurgeons; (**b**) 3D model preparation for printing; (**c**) 3D printed model before the removal of the support structures.

The unruptured R-MCA aneurysm 3D printed model of patient #2 showed a frontal branch wrapping the aneurysm (Figure 5), with no apparent gap between the artery and the aneurysm neck, which was not detectable with the initial 2D neuroimages. A multiple clipping was planned and then executed during surgery.

**Figure 6:**
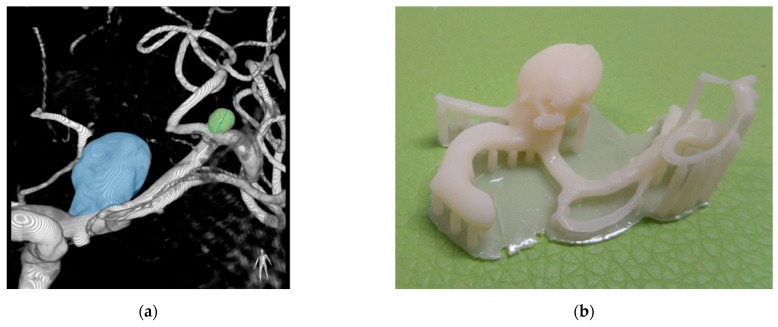

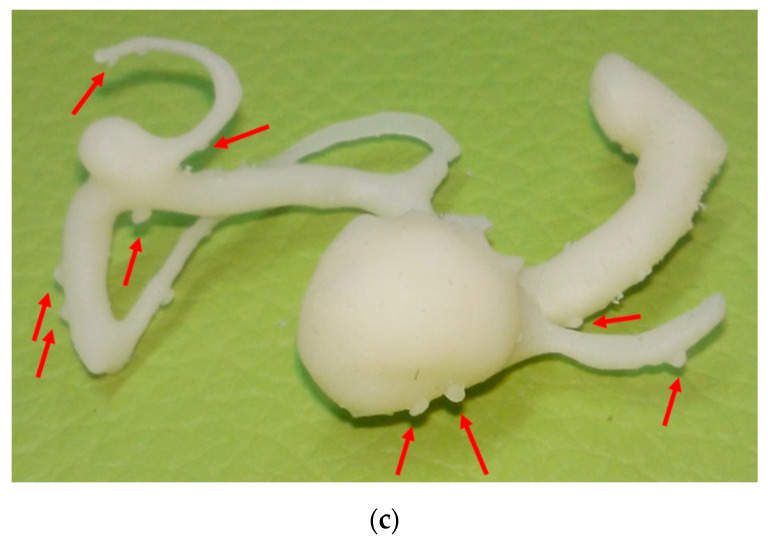
Patient #3 aneurysms reproduction and printing: (**a**) 3D reconstruction of DSA showing the L-ICA (light blue) and the L-MCA (light green) aneurysms; (**b**) 3D printed model showing the support structure before removal; (**c**) 3D printed model after the removal of the support structures (the red arrows indicate the positions of the supporting pins).

For patient #3, with two aneurysms (i.e., a L-ICA of 16 mm and a L-MCA of 4 mm), the 3D model showed a well demarcation between the neck of the aneurysms and the surrounding vessels (Figure 6); therefore, a simple clipping was planned. The intraoperative findings showed a well in the detachable vessels, and a single clipping was performed.

In general, the hardness of the models (see Table 3) did not allow a real clipping simulation, and the printed structure lacks a detailed and extended reproduction of the surrounding structures, such as the brain parenchyma, nerves, and bone. Moreover, this study focused on patient-specific cerebral aneurysms models, while using a generic approach for the fabrication of standardized validated models would allow comparability in surgical simulation and the possibility to obtain various versions of the anatomical model starting from only one [53].

Finally, the survey conducted with the 10 neurosurgeons who participated during the planning and the surgery activities to evaluate the usefulness of the 3D printed models as supporting tools for neurosurgery showed an average score of 4.5/5.

The neurosurgeons agree about the usefulness of the 3D printed models, which improved their understanding of the aneurysm’s relationship to the parent artery and of the surgical view in general.

## 4. Conclusions

In this study, 3D models of different cerebral aneurysms were printed through the direct light processing technique, resulting in a straightforward, easy to manage, and time- and cost-efficient process.

During the reconstruction of the 3D models, one of the main drawbacks is the lack of standardization of the starting DICOM files, especially with the CTAs, causing difficulties and inaccuracies in the STL elaboration, which, therefore, strongly depends on the experience of the operator who manually defines the contours of the slice images for the reproduction of the aneurysm structure. In fact, even if the software can automatically do this operation, some information can be lost and, therefore, the model cannot be considered representative of the actual anatomical structure. Moreover, the manual processing of the image files requires a longer time, i.e., at least twice the time of the automatic processing.

The mean total time from the elaboration of the STL file to the removal of the support structures necessary for printing was about 183 min and 170 min for the manual and automatic processing, respectively. These values can be considered very interesting and also absolutely suitable for emergency use in the case of ruptured aneurysms, which need around 150 min to be ready for surgery. In this way, the 3D printed model could be available for the neurosurgeons just before they reach the brain and the aneurysm site, thus allowing better planning of the clipping.

The average total cost per model is about €1.19, thus making this technology affordable and fully enterable in the patient management and in the neurosurgery learning and training programs.

In general, the hardness of the models did not allow a real clipping simulation, and the printed structure lacks a detailed and extended reproduction of the surrounding structures, such as the brain parenchyma, nerves, and bone.

During the neurosurgical application, the printed models turned out to be of practical support, helping the neurosurgeons to plan the right clipping approach with no residual neck of the aneurysms after intervention.

A qualitative survey about the usefulness of the 3D printed models conducted with the neurosurgeons who participated in the planning and surgery activities found that their application improved the understanding of the anatomy and vascular relationships of the aneurysm and surrounding vessels and improved surgery planning.

Future developments could relate to the possibility to print, in a time- and cost-effective way, hollow models with a consistency similar to the vessel one to further enhance the perception of the structure and to train regarding the application of the planned clipping.

## Figures and Tables

**Table 1:** Main information and characteristics of the patients and their cerebral aneurysms. L-MCA: left middle cerebral artery; R-MCA: right middle cerebral artery; L-ICA: left internal carotid artery.

Patient, #	Age, Years	Location	Dimension, mm
1	59	L-MCA	5
2	66	R-MCA	7
3	67	L-ICA/L-MCA	16/4

**Table 2:** Main characteristics of the DLP SHAREBOT Voyager 2 3D printer adopted for the realization of the 3D models of the cerebral aneurysms.

Characteristic	Value/Type
Printing volume, mm^3^	60 × 100 × 100
Light wavelength, nm	405
XY-resolution, µm	±50
Z-resolution, µm	5–100
Projector type	LED UV FULL HD
Projector resolution, px	1920 × 1080
Nominal power, W	40

**Table 3:** Main mechanical properties of the Share-HT photoactive resin used for the realization of the 3D models of the cerebral aneurysms. The values reported are those declared by the producer.

Property	ASTM	Value
Tensile strength at break, MPa	D-638	30
Young’s modulus, GPa	D-638	1.75
Elongation at failure, %	D-638	4
Shore D hardness	D-2240	77

**Table 4:** Time evaluation for the reproduction and printing of the 3D aneurysm models.

Patient, #	$t_{man}^{STL},\;\min$	$t_{aut}^{STL},\;\min$	$t_{P},\;\min$	$t_{S},\;\min$	$t_{man}^{Tot},\;\min$	$t_{aut}^{Tot},\;\min$
1	20	10	125	40	185	175
2	30	10	118	30	178	158
3	15	8	135	35	185	178

$t_{man}^{STL}$: time for the elaboration of the STL file by manually defining the images’ contours from the DICOM files; $t_{aut}^{STL}$: time for the elaboration of the STL file by automatically defining the images’ contours from the DICOM files; $t_{P}$ time for the printing process; $t_{S}$: time needed for the preparation and slicing of the 3D models within the slicing software and for the manual removal of the support structures; $t_{man}^{Tot}$: total time considering the manual elaboration of the STL file; $t_{aut}^{Tot}$: total time considering the automatic elaboration of the STL file.

**Table 5:** Cost analysis for the production of the 3D models of the cerebral aneurysms by using the DLP technology.

Item	Value
Printer investment cost, €	23,000.00
Raw material, €/kg	292.80
Energy consumption ^1^, €/kWh	0.2181
Average model volume, mm^3^	2.2 × 10^3^
Amortization period, y	5
Total cost, €/model	1.1868

^1^ For a small-sized enterprise with a total energy consumption between 20 and 500 MWh/y.

**Table 6:** Comparison of the literature results in terms of printing time and printing cost for the reproduction of cerebral aneurysms.

Printer	Material	Printer Cost, €	Printing Time, min		Model Cost, €		Reference
D-Force 400	PLA	510	100		0.35 ^1,3^	23 ^2^	[25]
UP! Plus	ABS	670	120 ^1^	>720 ^2^	1.25 ^1^		[38]
Objet500 Connex3	Rubber Shore A27	236,000	-		10		[48]
UP! Plus	ABS	670	>720 ^2^		1.15 ^1^		[49]
Prusa i3 MK3S	PLA	770	240		-		[50]

^1^ For the fabrication of the 3D model of the aneurysm and the anatomic structures in the immediate vicinity or in contact with it. ^2^ For the fabrication of the silicon-based hollow model of the cerebral aneurysm. ^3^ Considering only the cost of PLA.

**Table 7:** Comparison between the planned and the used approach for the treatment of the three patients’ aneurysms. The multiple clipping strategy is proposed for tightly adhered vessels to the aneurysm, while the single clipping strategy is for vessels that are fully detachable. For the characteristics of the patients, please refer to Table 1.

Patient, #	Estimated Clip	Used Clip
1	Multiple	Multiple
2	Multiple	Multiple
3	Single	Single

## Data Availability

Data sharing not applicable.
